# Framework for evidence synthesis of traditional, complementary and integrative medicine systems incorporating context and complexity, illustrated by the example of homeopathy

**DOI:** 10.3389/fpubh.2026.1752779

**Published:** 2026-04-01

**Authors:** Robbert van Haselen, Martin Loef, Pradeep Dua, Linda Zhong, Michael Teut, Janney Wale, Rajkumar Manchanda, Stephan Baumgartner

**Affiliations:** 1 International Institute for Integrated Medicine, London, United Kingdom; 2 Society for Clinical Research, Berlin, Germany; 3 Institute of Integrative Medicine, University of Witten/Herdecke, Herdecke, Germany; 4 Traditional, Complementary, and Integrative Medicine (TCI) Unit, Department of Integrated Health Services (IHS), World Health Organization (WHO), Geneva, Switzerland; 5 Biomedical Sciences and Chinese Medicine, School of Biological Sciences, Nanyang Technological University, Singapore, Singapore; 6 Independent Researcher, Berlin, Germany; 7 Independent Researcher, Brunswick, VIC, Australia; 8 Department of Medicine, Government of Delhi, Nehru Homoeopathic Medical College and Hospital, New Delhi, India; 9 Institute of Complementary and Integrative Medicine, University of Bern, Bern, Switzerland

**Keywords:** complex systems perspective, evidence framing policy implications, evidence synthesis, homeopathy, TCIM, whole medical systems

## Abstract

**Background:**

Evidence synthesis for whole medical systems, defined as complete systems of theory and practice that have evolved independently from biomedicine. is challenging. This paper provides a framework for evidence synthesis of Traditional, Complementary and Integrative Medicine (TCIM) systems incorporating context and complexity. A systematic review program on the effects of homeopathic preparations is used as a practical illustration of the proposed framework.

**Proposed solution:**

A systems perspective considers the patient as a complex system of interconnected regulative subsystems embedded in a complex environment, and disease as a dysregulation of the dynamic adaptive state of the organism. Most TCIM systems, including homeopathy, aim to stimulate regulative systems and their functions to regain homeostasis. The consequences of these principles for the approach to evidence synthesis of TCIM systems are explored and explained.

**Framework components:**

A systems perspective takes into account a plurality of evidence sources, including ‘real-world’ clinical data such as case reports, case series and cohort studies. The systematic review program focuses on comparative studies of homeopathy in various clinical indications and includes both non-randomized prospective studies of interventions (NRSIs) and randomized controlled trials (RCTs). For risk-of-bias assessments, use is made of respectively ROBINS-I and ROB2. Evidence certainty is graded transparently and rigorously based on the GRADE framework. An experienced patient advocate is involved in the research program and input from patient advisors who experienced the clinical indication under investigation is incorporated.

**Discussion:**

RCT and NRSI evidence on homeopathic preparations for a range of clinical indications will be synthesised. A limitation, from a complex systems perspective, is that the implication of these findings will still need to be contextualized within the broader context of the existing state of knowledge. An ‘evidence eco-system’ that includes complementary sources of information will be required to inform decisions.

**Conclusion:**

Evidence synthesis of TCIM systems can move beyond conventional approaches by framing evidence within its complexity and context, together with real-world data and patient perspectives. This approach entails methodological challenges and will require gap analyses to guide future research and improve the applicability for public health and individualized patient care.

## 1 Introduction

Homeopathy is part of a broad group of therapies, referred to as Traditional, Complementary and Integrative Medicine (TCIM) (1), or Traditional, Complementary and Integrative Healthcare (2). It was founded more than 200 years ago as a European Traditional Medicine system by the German physician Samuel Hahnemann, as a personalized system of medicine which aims to stimulate patients’ natural healing capacities. The clinical effects of homeopathic preparations (in the European Union also referred to as homeopathic medicinal products) as used in homeopathy and anthroposophic medicine have been scientifically debated for more than a century. The most recent systematic review of 6 meta-analyses concludes that the available data on the effects of homeopathic preparations in any indication are not compatible with the placebo hypothesis (3). This conclusion was maintained in sensitivity analyses restricted to the meta-analyses with a low risk of bias and in sample restrictions based on inclusion of only high-quality trials.

While many systematic reviews of TCIM systems have been published in the last few decades, there are still insufficient indication-specific systematic reviews (including meta-analyses) that comprehensively evaluate the literature in accordance with contemporary scientific standards while simultaneously incorporating the complexity of these therapeutic systems. The need to address this gap is also recognized as important by the WHO in its 2025–2034 global traditional medicine strategy (4). This strategy propagates acknowledgment of the unique characteristics of TCIM systems and calls for the further development of innovative approaches incorporating complexity science and real-world data.

Within the broad group of TCIM modalities, the US National Institutes of Health distinguishes ‘whole medical systems’, the main ones being traditional Chinese medicine, ayurveda, Unani, homeopathy, naturopathy and anthroposophic medicine (5). A recent, more elaborate operational typology involving six main classes of TCIM approaches is proposed by Ijaz (6): (A) orally transmitted ethnomedical systems and practices; (B) codified ethnomedical systems and practices; (C) non-ethnomedical whole systems; (D) complementary therapeutics; (E) community-based therapeutics; and, (F) integrative therapeutics. This classification is compatible with the above-mentioned WHO 2025–2034 traditional medicine strategy, in which the distinction is made between codified and non-codified traditional medicine systems. In this article we principally refer to codified ethnomedical (e.g., traditional Chinese medicine and ayurvedic medicine) as well as non-ethnomedical whole systems (e.g., homeopathy and anthroposophic medicine). For the sake of convenience, the terms TCIM and whole medical systems are used interchangeably, with the clarification that the principal, but not exclusive focus is on individualized, whole medical systems.

With the rise of evidence-based medicine (EBM) as a movement in the 1980s, the topic of complexity was initially ignored. The focus was on investigating specific effects of interventions in randomized controlled trials (RCTs). Narrowing the focus of RCTs had (and has) the advantage of reducing ‘unwanted variability’ (‘noise’) in RCTs. Moreover, randomization was (and is) a smart way of dealing with the problem of complexity; if well implemented, it maximizes the likelihood that both known and unknown individual determinants of outcome (confounders) are equally distributed between the treatment and control groups, enabling internally valid comparisons. Therefore, randomization effectively avoided having to deal with the challenge of complexity. This may have contributed to a lack of emphasis on the ‘contextualization’ of findings, as also argued by experts from within the field of EBM (7–9).

By the end of the 20th century, it was becoming increasingly clear that many interventions often interacted in non-predictable ways with elements of the healthcare systems in which they were embedded. Raising awareness of the importance of factors related to the context of healthcare systems led to a first workshop on complex interventions at the 1994 Cochrane Colloquium with a report published the next year (10). A complex interventions perspective locates sources of complexity in the features of interventions themselves, emphasizing multi-causal, rather than mono-causal, pathways. The first detailed guidance on complex interventions was published in 2000 (11). A second perspective is a ‘complex systems perspective’, which locates the sources of complexity in the properties of systems as a whole, including patients as ‘biological systems’, into which interventions are introduced (12). Complexity as a concept is underpinned by theories that are used to understand the dynamic and adaptive nature of interventions and systems (12, 13). Therefore, in medicine, the term ‘complexity’ can refer to the complexity of the intervention, the complexity of the systems—the patients—that are the target of the intervention, as well as the complexity of a framework for conceptualizing and analyzing a problem.

During the last few decades, the topic of complexity has been elaborated further in the systematic review community, resulting in an on-going stream of publications, including a continuously updated section on intervention complexity in the Cochrane Handbook (14). In parallel, complexity in a broader sense has become a burgeoning domain in the natural sciences (15) as well as in the social sciences and in philosophy (16).

Inspired by ontological and epistemic similarities, the TCIM community recognized the potential relevance of complexity science for the study of the effects of TCIM modalities (17–21). Homeopathy was at the forefront of these developments (22, 23). Subsequently, complexity science has been proposed as a possible framework for explaining the principle of similars (one of homeopathy’s core principles) (24), possible effects of low-dose nanoparticles (25, 26), the complexity of the homeopathic healing response (27, 28) and also for phenomena observed in clinical trials (29, 30).

Homeopathy is a complex, multicausal, patient-centered, whole medical system (5). This complexity is expressed at the level of the intervention as well as the patient as a complex adaptive system. The treatment setting in which most homeopathic preparations (HPs) are administered is characterized by therapeutic individualization, and also influenced by non-specific effects that are part of the patient-therapist relationship (31). Since a key principle is to trigger an adaptive reaction in the patient, rather than to exclusively treat the clinical diagnosis, systemic and/or non-linear effects can occur (5). According to the latest Cochrane guidance (14), systemic and non-linear effects should be taken into account when synthesizing evidence in systematic reviews. However, this has not been instigated yet, or only to a very limited extent, in systematic reviews of whole medical systems.

Since the publication of the seminal review of clinical trials of homeopathy by Kleijnen et al. (32), several more reviews have been conducted, some of which reached positive conclusions (33–35) and some of which did not (36, 37). The currently most comprehensive reviews (38–41) suggest a superiority of homeopathy over placebo (3), especially if the selection of the HPs was carried out according to basic homeopathic principles.

However, these reviews have reduced the discussion around homeopathy to a simple “is it a placebo yes or no” question. By contrast, a complexity perspective requires addressing context related questions such as when, why, how and in what circumstances interventions work well (42, 43). A recent exhaustive literature screening conducted in preparation for this project (44) identified a significantly higher number of randomized controlled trials (RCTs) compared to those included in the above-mentioned reviews of homeopathy.

Against this background, the German Society for Clinical Research (Gesellschaft für Klinische Forschung, Berlin) has embarked on a program (under the acronym HOMA) of up to 12 systematic reviews with or without meta-analyses that aims to address this gap. The present publication aims to provide the broader context in which the HOMA project is embedded, which highlights the importance of complexity, context and patient perspectives, as appropriate for many Traditional, Complementary and Integrative Medicine (TCIM) systems. This framework consists of various interconnected levels; the ‘highest’ level is the societal context in which the evidence is applied; the next level describes TCIM from a complex systems perspective and elaborates on the implications for evidence requirements. A brief overview of the literature on incorporating complexity in evidence synthesis is provided. It is explained that the latter requires a mapping and visualization of the complexity of the TCIM modality concerned. Furthermore, it is recommended to provide a conceptual framework for the purported mode of action of the TCIM modality concerned. The next level addresses the implications for systematic review protocols of TCIM, using homeopathy and the protocol template of the HOMA systematic review program^1^ as an illustration. The final, most focused, level involves tailoring the systematic review protocol template on a TCIM specific basis to the therapeutic indication. This paper reviews, and provides a framework for, evidence synthesis of Traditional, Complementary and Integrative Medicine systems incorporating context and complexity. The HOMA systematic review program is used as a practical illustration of the proposed framework.

## 2 Objectives

To approach TCIM systems in general, and homeopathy in particular, from a complex systems perspective. This will appropriately frame the systematic review program, guide the analysis and interpretation of findings and help formulate hypotheses on why, how, when and where HPs are effective.

To enable and facilitate the development of a comprehensive, pluralistic framework, in which there are complementary types of evidence within a broader ‘evidence ecosystem’.

## 3 Framing approach

Complex systems thinking is characterized by an emphasis on multiple interacting, interconnected networks, not only within one level in the system, but also between different levels, like for instance in an eco-system. An example of applying systems thinking to biomedicine is the description of “health” as ‘the successful homeostatic integration of different regulative circuitries, from the molecular level to the whole organism’ (45).

An analogous approach regarding the framework for TCIM evidence synthesis is employed. In this publication, reference will be made to TCIM evidence synthesis in general and practically illustrated based on the framework for the homeopathic systematic review and meta-analysis program (the acronym ‘HOMA’ is derived from ‘Homeopathy’ and ‘Meta-Analysis’).

The different levels in the framework are described, including how these levels interact with each other, with particular emphasis on aspects that are relevant for guiding the scope and nature of systematic reviews of TCIM modalities. The practical implications of the chosen framing are illustrated by the HOMA project systematic review protocol template.

This approach is in line with, and inspired by, Chapter 17 of the Cochrane Handbook (14), which argues that complexity should be considered as a multidimensional continuum, where there may be higher or lower levels of complexity across different aspects of the intervention and for those involved in delivering or receiving it.

## 4 Overview of the framework for evidence synthesis of TCIM systems

**Figure 1:**
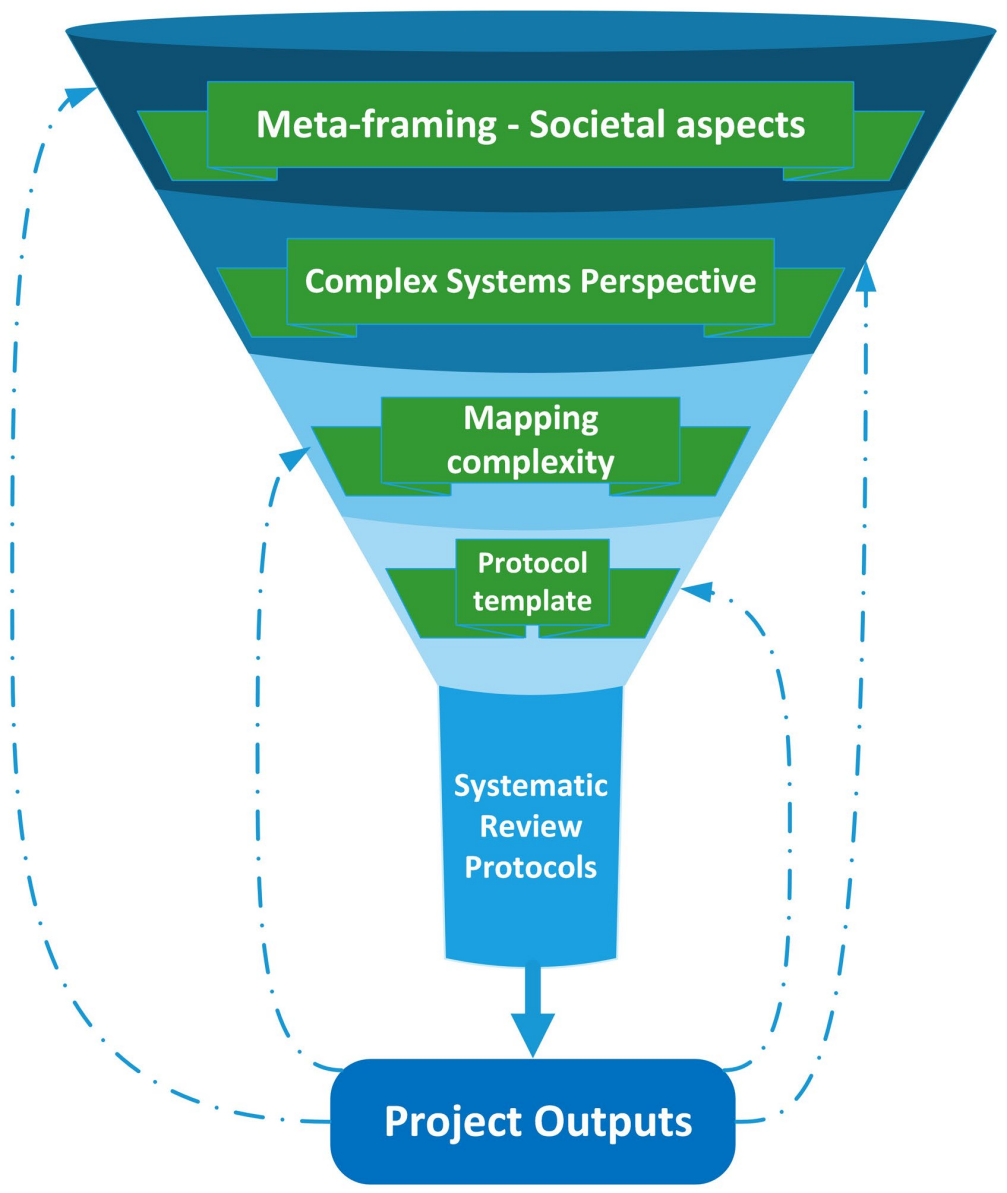
Overview of framework for whole medical system systematic review programs.

The framework is visualized in Figure 1.

As indicated in Figure 1, the overall framework consists of various levels, ranging from societal ‘meta-framing’ dimensions as the broadest level, to the specific systematic review protocols as the most focused and detailed level.

Examples of complexity focused review questions are given in Box 1.

Box 1 Examples complexity related review questions.

- What overarching societal or policy goals should the evidence synthesis address?
- How do TCIM systems relate to the health system as a whole?

Complex systems perspective

- What feedback loops or interactions between intervention, context and outcomes can be expected?

Mapping complexity

- Which contextual factors (e.g. patient characteristics, cultural attitudes, care pathways) influence effectiveness?

Protocol template

- Which elements need to be systematically captured to reflect context and complexity?

Protocol

- How will heterogeneity and context sensitivity be addressed?

In general, the broader framing levels provide context for, and inform, subsequent more focused levels. At the same time, project outputs provide feedback for all the levels of the framework. On the one hand, results of TCIM systematic review programs will inform societal discussion about the role of TCIM in healthcare systems; on the other hand, each systematic review is likely to lead to improvements in the protocols of subsequent systematic reviews. Interactions between systematic review results (the products) and dimensions of the process that generated these results are in themselves a reflection of ‘complex systems thinking’, which emphasizes a circular—rather than a linear—perspective on causality (46). It also underlines the core principle that findings should always be reported and reframed in the broader context of the higher levels of the framework.

## 5 Elaboration on elements of the framework

### 5.1 Meta-framing

The rationale behind the HOMA project is societal demand for Traditional, Complementary and Integrative Medicine (TCIM) in general, and the continuing demand by patients for homeopathic (47, 48) and anthroposophic care (49) in particular. From a public health perspective, this societal demand is reflected in existing regulatory frameworks for homeopathic practice and products in many countries. The latter includes the manufacturing of HPs in accordance with recognized homeopathic pharmacopeias, such as the European Pharmacopoeia.

A key aspect that many TCIM modalities have in common is a research strategy that is the ‘reverse’ of the research strategy for the development of new chemical entities in biomedicine. The flow of the research data generation in biomedicine is ideally (and therefore not always) based on a pathophysiological rationale “from the (laboratory) bench to the bedside”. The situation for TCIM modalities is the reverse; the basis of the intervention is developed over long-term clinical practice through iterative observation and experience. It is often only *after* the development of the medical systems, sometimes over thousands of years like in ayurvedic medicine and traditional Chinese medicine, that clinical trials and laboratory investigations as methods come into the picture (50, 51). This aspect is also referred to as a ‘reverse research strategy’ or ‘reverse pharmacology’ (52) and is part of the meta-framing of the HOMA project.

Systematic reviews are produced and used in the broader context of what has been described as an ‘evidence ecosystem’ (53). This ‘systems perspective’ on the systematic review process itself is characterized by an emphasis on the conduct of multicomponent reviews that use different types of evidence (54), and by mixed treatment comparisons in network meta-analyses. What and how much evidence is required depends on broader aspects of this evidence ecosystem, which is relevant in the context of TCIM.

As mentioned above, TCIM modalities have a ‘reverse research strategy’ compared to the research strategy for biomedicines. One implication of this is that observational clinical data, as generated during decades or centuries of traditional use, are much more common than clinical trial data and preclinical laboratory data (50, 51).

The high public demand for homeopathy in many countries is reflected in a societal need for a risk-based regulatory framework (55) in which the importance of ensuring both the availability of HPs as well as the safety of patients and users is paramount. According to the Oxford English Dictionary, ‘risk’ refers to the ‘chance or possibility of danger, injury or other adverse consequences’ (56), and it should be pointed out that this is a broader concept than adverse reactions to drugs: The clinical risk associated with an indication claim is determined by the risk for a patient if the intervention is ineffective. For instance, the low risk associated with the indication-claim that a HP ‘alleviates symptoms of the common cold’, is clearly different compared to the high risk associated with the claim that a HP “cures pneumonia.”

In brief, the context in which the evidence is applied will influence the required evidence standards, and thereby the acceptable level of uncertainty in recommendations from systematic reviews. This aspect is not TCIM specific; in biomedicine the extent and quality of evidence required will also depend on the context in which decisions have to be made. For instance, during the COVID-19 pandemic, pressing decisions had to be made either based on limited evidence, or based on no evidence at all. A lack of appreciation for the influence of context factors on the required evidence standards is a major drawback for medicine in general, and TCIM in particular.

A key message is that the evidence requirements for TCIM modalities are not absolute; what is an acceptable level of certainty will depend on the complex interaction between product safety, history of use and the risk level of the indication claim. If the overall risk level is low, existing or new data that provide requisite certainty can be acceptable, while a higher level of certainty (of evidence) is required if the overall risk level is moderate or high. Encouragingly, frameworks that aim to navigate uncertainty in evidence informed decision-making in TCIM are under development (57).

### 5.2 Complexity: implications for medicine and evidence requirements for TCIM modalities

While the word ‘complexity’ is etymologically related to the word ‘complicated’, it is important to point out that these terms have distinct meanings (58). For instance, objects such as watches, other machines or computers are ‘complicated’; though composed of many parts, a complicated system behaves in a predictable, deterministic way. Complexity in humans and many higher animals is characterized by the occurrence of nonlinear interactions under certain circumstances, leading to non-deterministic behavior of the system. This is illustrated in homeopathy by the expectation of context-dependent non-linear effects; i.e. the patient is only expected to respond when the homeopathic preparation is correctly matched to the patient’s constitution and/or symptomatology.

More generally, outcomes can be influenced by various contextual factors that serve as ‘triggers’. Complexity is generated by the interactions between multiple contextual factors and mechanisms that contribute to an outcome (59).

### 5.3 Complexity in TCIM

We will explain what a complex systems perspective involves, including why and how it is relevant for medicine, as well as for TCIM in particular. For clarification, TCIM entails ‘Integrated’ medicine or healthcare which, as stated in the “People’s Declaration on Traditional, Complementary and Integrative Healthcare,^2^ involves “Integrating traditional, complementary and biomedical practices in a supportive and collaborative manner.” This is why in this section we also refer to ‘medicine’ under the header of TCIM.

A complex systems perspective on medicine takes into consideration the dynamic properties of the context into which a medical intervention is introduced (12) as well as the ways in which processes and outcomes at all points within the healthcare setting drive change. A complex intervention is characterized by a focus on (A) possible interactions between the components of the intervention, (B) interactions between the intervention and the healthcare system in which it takes effect, (C) adaptive behavior in response to changes in the environment, of which the therapeutic intervention is one, (D) non-linear effects, (E) emergence of new properties, (F) amplifying and inhibiting feedback loops, and (G) multiple outcomes and dependencies.

A systems perspective can be adopted at the level of the individual patient, as well as at the population and/or healthcare system level (60). In a systems perspective, as Aristotle argued, the whole is more than the sum of its parts, and in this sense it involves a holistic way of seeing the world (61). It is therefore unsurprising that this perspective resonates strongly in the TCIM community. In whole medical systems, disease is considered to be a disequilibrium between biological, psychological, social and spiritual forces (5, 62). The patient is understood as a complex system of regulative subsystems, and disease as a dysregulation in the dynamic adaptive state. Correspondingly, health is characterized by a dynamic stability of the complex system within defined boundaries, as maintained by an organizing principle. In TCIM systems this organizing principle is for instance referred to as ‘qi’ in Traditional Chinese Medicine, as ‘prana’ in Indian Medicine and the founder of homeopathy referred to it as the ‘dynamis’ and the ‘vital force’ (63).

Also, ‘Gestalt’ theory, which emphasizes a holistic and dynamic organization of elements, can provide insights in medical processes from a systems-theoretical perspective. Gestalt theory was developed by Karl Duncker (64) who defined a ‘Gestalt’ as a physical, biological, or symbolic configuration of elements, whose properties cannot be fully explained by the sum of its parts. The Gestalt ‘comes into being’ or, to use complex systems language ‘emerges in a non-linear way’, from the parts. In Gestalt therapy, the healing process is viewed as a temporal and spatial gestalt transformation, with changes in its elements analyzed as part of this evolving structure. Like in a complex systems perspective, this allows medical interventions to be seen and understood as interactions within interconnected systems, highlighting the emergent effects of element interactions and the self-organizing capacities of both patients and treatment contexts.

In many TCIM systems treatment is ‘personalized’ with the aim to trigger (ideally in a catalytic way) the patient’s innate self-organizing mechanisms. For example, acupuncture in Traditional Chinese Medicine works precisely by stimulating and promoting the body’s self-healing capacity. It guides, amplifies, and accelerates the body’s inherent regulatory and repair processes through physical stimulation such as with acupuncture needles and moxibustion (65).

In Ayurvedic medicine, treatments are similarly personalized with the aim of catalyzing the body’s innate self-regulatory and healing capacity. For instance, *Panchakarma* therapies (66) such as *Virechana* (therapeutic purgation) or *Basti* (medicated enema) are designed to clear accumulated toxins and reset the body’s natural homeostasis, thereby stimulating its capacity to restore balance. *Rasayana* (rejuvenation) therapies, including formulations like *Amalaki* (67–69) or *Ashwagandha*, work to activate intrinsic repair and regeneration processes, supporting vitality and resilience. Likewise, individualized dietary and lifestyle prescriptions based on a person’s *prakriti* (constitution) (70–72) aim to harmonize digestion and metabolism, while practices such as yoga and pranayama help regulate psycho-physiological balance, further amplifying the body’s inherent healing mechanisms.

This is also reflected in homeopathy, in which the main cause of the healing process is assumed to come from within the system, with the administered HP serving as a trigger (73); to use complex systems language (74): ‘HPs trigger events in systems’.

In TCIM, the focus is therefore not on the intervention as ‘external’ to the system, rather it is on the dynamic properties of the patient—as a complex system—to which the intervention is introduced (12). From a complex systems perspective, desirable outcomes are not limited to clinical indicators but also include emergent properties such as strengthened adaptive capacity, improved patient–practitioner relationships, and sustained shifts in mental, emotional and physical functioning. For example in homeopathy, these concepts are described in detail in the ‘levels of health’ theory (75). Outcomes such as overall symptom reduction, patient satisfaction, higher levels of energy, etc., may be interpreted as markers of successful system adaptation. However, these outcomes are rarely captured in homeopathy studies, representing both a gap in the evidence base and a priority for future research.

In biomedicine, the adaptive reaction of the patient is sometimes desired (e.g., vaccines), but often undesired (i.e., ‘side-effects’ of the drugs, e.g., systemic reactions to antibiotics and cytokine storms with immunotherapy). In complex systems, adaptive responses tend to be non-linear due to amplification of positive and/or negative feedback loops. In conventional pharmacology, linear dose–response relationships are usually considered to be desirable. From a systems perspective, linearity is the ‘limit case’ of non-linearity and can be expected after exposure to strong external stimuli that predominate (e.g., conventional medicines acting as external agents directly on specific molecular receptors in the patient), overwhelm (e.g., toxins with noxious biological effects) and/or bypass (e.g., opiates passing unhindered through the blood–brain barrier) the patients’ adaptive capabilities.

In biomedicine, there is a trend toward increasing ‘personalization’ of treatment (76). However, the emphasis is on genetic profiling and identifying molecular markers to identify patients with a particular diagnosis that are more likely to respond to a particular medicine. Therefore, the ‘personalization’ of the treatment involves a more targeted treatment of the disease as a diagnostic, pathophysiological entity. This is different from, and should not be confused with, a complex systems perspective; the latter emphasizes the role of self-organization of the whole system in sustaining and maintaining health.

**Figure 2:**
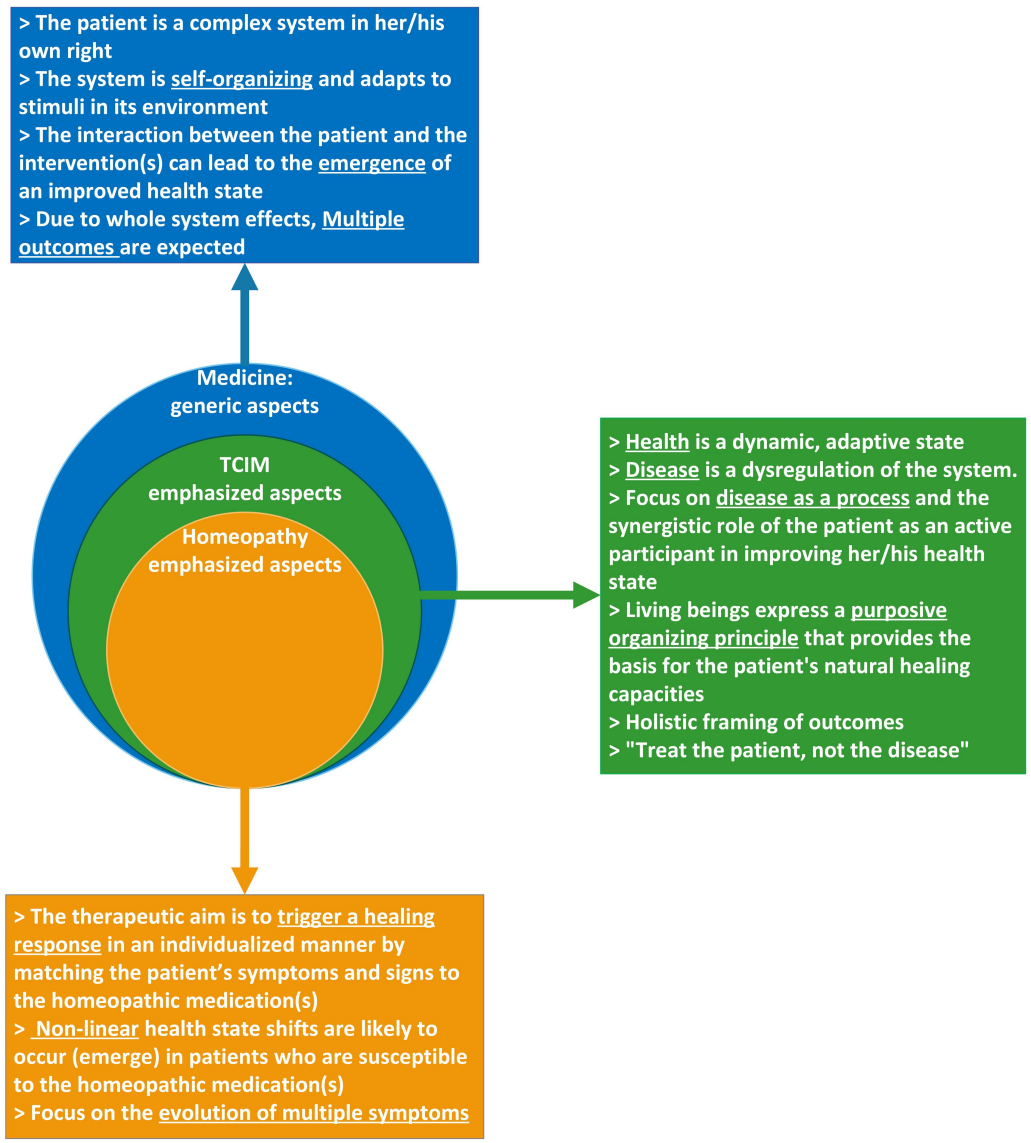
Systems perspective overview, detailing aspects that are emphasized in TCIM and in homeopathy.

Figure 2 summarizes and visualizes key aspects of the complex systems perspective as referred to above and in Table 1 below. It differentiates those aspects that are applicable to medicine in general (in blue) from those that are emphasized in TCIM systems (in green), and in homeopathy in particular (in orange). Conceptually, the homeopathic aspects (orange circle and text box) are contained within the TCIM aspects (green circle and text box), and both are contained within the blue circle of a complex systems perspective on medicine in general (blue circle and text box).

**Table 1:** Characteristics of a coplex systems perspective on medicine in general, as well as specific aspects that are emphasized in TCIM and homeopathy, with some examples of suitable evidence types and research designs.

Medicine in general; core assumptions	TCIM as whole systems orientated approaches; emphasized aspects	Homeopathy as a specific TCIM system; emphasized aspects	Description of suitable evidence and research designs to address this aspect (non-exhaustive list)	Examples
The patient is a complex system in its own right. Characterizing features of a systems perspective on clinical practice are: individualized treatments, minimized interventions, multidimensional use of medications, time-sensitive treatments, synergistic combination treatment, temporal treatments and probabilistic forecasting of success or failure of individual treatments	Health in a patient is considered to be a dynamic state / process. It is characterized by the ability to adapt and self-manage in the face of social, physical, and emotional challenges (140) Disease is characterized by a short term (acute) or long term (chronic) dysregulation of the system. It can be triggered by an accumulation of various (noxious) external and/or internal stimuli Symptoms are considered to be the most intelligent responses by the system following an internal or external perturbation to which the organism is susceptible Clinical practice and experience are the basis of the knowledge generation process	Homeopathy emphasizes the role of all the symptoms and signs as experienced by the individual patient as the unique way in which the system expresses its dysregulated state Symptoms and signs have a central and positive signaling function for guiding the appropriate intervention Clinical symptoms (pathophysiologically related to the clinical diagnosis) as well as NON-clinical symptoms (experienced only by some patients and NOT pathophysiologically related to the clinical diagnosis) are considered to be important for guiding the selection of the HMP	Contrary to the situation for conventional medicines, the flow of the knowledge generation process is ‘from the whole to the parts’ (51), i.e., from effectiveness and safety in clinical practice as assessed in case reports, case series and single-arm cohort studies (141), to understanding the role of context and the individual patient, to non-randomized and randomized comparative effectiveness studies, to component efficacy and biological mechanisms More emphasis on a ‘mosaic’ of research methods, than on an evidence hierarchy with RCTs at the top. More emphasis on ‘real-world effectiveness’ than on efficacy in highly selected, non-representative-patient populations	Traditional Chinese Medicine emphasizes the interaction of time, location, space, and people (142) The safety of many HPs is supported by a long tradition of use Patient satisfaction is supported by survey data The plausibility of ‘real world effectiveness’ is supported by comparative pharmaco-epidemiological studies (143, 144)
Self-organization: The dynamics of a system arise spontaneously from its internal structure in a self-generating (“autopoietic”) manner. Health and disease are assumed to be dynamic ‘attractor states’	Living beings express a purposive organizing principle that can be utilized as the basis for the patient’s natural healing capacities	The aim of HPs is to stimulate the natural healing capacities in susceptible patients in an individually targeted manner.	Qualitative and quantitative research methods to obtain an in depth understanding of the factors that influence the susceptibility of patients to healing stimuli.	Hahnemann proposed the conduct of homeopathic pathogenetic trials (“provings”) to understand the effects of HMPs on healthy subjects, with a view to identifying which HPs can have healing effects in which patients.
The patient *adapts* to changes in her/his environment, including the introduction of an intervention	The principal aim of therapy is to achieve or enhance the healing (adaptive) response in the patient	The principal aim is to achieve a healing response in an individualized manner by matching the patient’s symptoms and signs to the homeopathic medication(s) prescribed.	Allowing unrestricted administration of any required individualized HPs in RCTs Identification of prognostic factors via the application of Bayesian statistics in clinical practice	Meta-analysis of all RCTs with individualized HPs as compared to placebo (145) Prognostic factor research was able to identify which HPs are more likely to be effective in which types of COVID-19 patients (146)
The *interaction* between the patient and the therapist and/or interventions can lead to the *emergence* of an improved health state	Disease and health are considered emergent properties of a person in her/his environmental context (62) Focus on disease as a process, rather than a discrete entity / state. Dynamic interactions within the patient, and between the patients and her/his environment are responsible for the overall health/disease state. In addition to addressing the main complaint, there is an interest in the patient as a whole. Emphasis on the role of the patient as an active participant in improving their own health state.	Administered HPs aim to trigger the emergence of an improved health state which is sometimes characterized by shifts in the symptoms and signs, in line with defined principles, such as, e.g., improvement in the opposite order of the development of the disease process	Due to the non-predictable and non-deterministic nature of emergent health states, clinical case reports can be useful as hypothesis generating tools	In a patient with severe neutropenia treated with individualized HMPs, the symptoms improved in the opposite order of their development (147) Homeopathy appeared to have a positive effect in the management of a life-threatening and intractable case of walled-off pancreas necrosis (148) Case series indicating enhanced healing in patients with diabetic foot ulcers (149) A patient with nephrotic syndrome was treated with natrium sulphuricum and followed-up for a year (150)
Due to the amplification of positive and/or negative *feedback loops*, emergent properties are *non-linear*	The emergent properties of the desired improved health state is ‘susceptibility dependent’ and non-linear.	Non-linear shifts in health states are likely to occur probabilistically in susceptible patients receiving optimally ‘matched’ HMPs. Non-linearity could be part of the explanation why the low doses applied in homeopathy can have clinical and biological effects. ‘Non-matched’ HPs are expected to have no effects, due to the highly diluted nature of most HPs. Treatment responses are principally substance dependent, and less dose-dependent.	Changes in health states following the administration of HMPs can be assessed in principle using all research designs. In RCTs and NRSIs, the effects of HMPs are expected to be underestimated or absent if some or all patients do not receive optimally ‘matched’ HMPs. Model validity as well as the skill and experience of the therapist are therefore potential confounding factors in RCTs and NRSIs.	Traditional Chinese medicine emphasizes the mutual support and restraint among the various zang-fu organs in the human body. Health is achieved only when a dynamic balance is maintained among them. Conversely, any excessive strength or weakness in the function of an organ can lead to problems in related systems (151) The importance of initiating non-linear shifts in homeopathic patients is reflected in the principle of the ‘minimum dose’: in order to reduce the likelihood of side-effects, only enough medicine should be employed to trigger the intrinsic healing response of the patient (152)
*Multiple outcomes* and dependencies in pathological processes are expected	The primary framing of outcomes is holistic, not just symptom based “treat the patient, not the disease”	Focus on evolution of all the symptoms, including etiological and pathological processes, in the patient as a whole (153)	Research designs and evidence syntheses should incorporate all or most of the outcomes into an integrated/synthetic outcome assessment that considers the evolution of the health state of the patient as a whole. The expected changes in individual outcomes are not fully pre-definable nor predictable.	It is now recognized in biomedicine as well that seemingly single phenotypic entities, e.g., various types of cancers and diabetes, can have multiple etiologic or pathological processes. This encourages the notion of more personalization and individualization in medicine in general.

### 5.4 Implications of a complex systems perspective for evidence requirements in TCIM

In this section we will elaborate on the implications of this framework for suitable evidence types for TCIM and illustrate this by some examples. It emphasizes the importance of an ‘evidence ecosystem’, rather than a narrow focus on the RCT as the main evidence type at the top of an evidence hierarchy.

Table 1 provides an overview based on available guidance publications (60, 77, 78), but with adaptations. It expands on aspects referred to in Figure 1 and provides some examples of evidence types and research designs that address these aspects.

It should be mentioned that the boundaries between the emphasized aspects are neither absolute, nor sharp. For instance, even though homeopathy emphasizes all the symptoms and signs as experienced by the patient, there can be an emphasis on both the pathophysiological (clinical) as well as other individual symptoms and signs as an expression of the dysregulation of the patient’s dynamic state. While the table emphasizes non-RCT evidence and points to instances where RCT evidence is suboptimal, it should be underlined that robust comparative designs are an important part of, and can be used to support, complexity-based approaches. For example, placebo-controlled RCTs can, while Non-Randomized Studies of Interventions (NRSIs) cannot, distinguish whether outcomes arise from the HP itself or from the consultation and care context. In conclusion, a complexity informed framework requires a methodological pluralism.

### 5.5 Complexity in evidence synthesis: an overview

For an overview of the implications of a complexity perspective for systematic reviews in medicine, see Petticrew et al. (60). Generally speaking, the application of a complex systems perspective in evidence synthesis is not yet well established (78). Moreover, almost all publications that provide guidance on complexity focus on (1) intervention complexity (i.e., multiple, identifiable components of the intervention), (2) causal pathway complexity, (3) implementation complexity, or (4) contextual complexity associated with the dynamic, multi-dimensional healthcare system environment. A fifth domain, population complexity, occurs when variant characteristics of the individuals, groups or organizations receiving the interventions modify the effects of the interventions (79). In principle, the latter domain includes the possibility that differences in individual patients modify the effects of the intervention, which theoretically brings it close to the core tenet of treatment individualization in TCIM. However, the focus in the available literature is mainly on general individual characteristics, such as co-morbidities or socio-economic class as effect-modifiers. By contrast, in homeopathy the concept of effect-modification is linked to individual patient characteristics as HP-related prognostic factors (80, 81). In homeopathy, these prognostic factors are the patient’s symptoms and signs as predictors for a positive response to administration of the individually tailored HP. This is also one of the reasons why in the HOMA project we have chosen to approach complexity from the perspective of the individual patient.

An important observation is that the existing literature on complexity has insufficiently addressed the perspective in which patients are considered as complex systems (‘population complexity’ as referred to above). A possible explanation for this is the wide adoption of randomization as a tool that balances prognostic factors, mitigating the necessity to deal with individual patient response variability. In TCIM the focus is on individualizing treatment based on the total symptom picture (5).

Investigating the complexity of interventions in evidence synthesis necessitates methodologies that account for interdependencies, contextual variability, and non-linear effects. Beyond tools like iCAT_SR (82) as adopted by Cochrane, realist reviews offer a theory-driven approach that elucidates how, why, and under what circumstances interventions produce outcomes. In this approach, use is made of a ‘Context–Mechanism–Outcome (CMO)’ framework to explain variability in outcomes (83). Complexity analysis can be further enriched by network-based approaches; for instance, network meta-analysis enables indirect comparisons across multiple interventions, and component network meta-analysis disaggregates complex interventions with the aim of assessing the contribution of individual elements. For a full discussion on the distinct assumptions, strengths, and limitations of each approach, see publications by Tsokani and Ades (84, 85). These approaches presuppose specific methodological and data-related conditions – such as adequate contextual reporting, assumptions of transitivity and network connectedness, and, in the case of component models, plausible additivity. Given the expected heterogeneity of interventions and study designs, their applicability requires case-by-case assessment. Where conceptually and empirically justified, they may provide complementary analytical perspectives in individual reviews. In contexts of sparse evidence, Bayesian modeling strategies may be considered, whereas frequentist multilevel models provide a pragmatic basis for sensitivity analyses. The choice between approaches will be guided by empirical feasibility and inferential coherence.

Mixed methods reviews, that use a variety of quantitative and/or qualitative evidence sources, can provide insight into context driven adaptation of TCIM interventions (86). Qualitative approaches can complement quantitative evidence synthesis by addressing dimensions such as for instance stakeholder perspectives, perceived mechanisms, and implementation processes (87).

In TCIM, tension is likely to remain between high levels of heterogeneity in the treatment approaches, and the need for some standardization of treatment that is required for the types of analyses referred to above. TCIM is likely to require different and complementary methodological approaches that preserve their integrative and emergent properties and improve the context-sensitive understanding of intervention complexity in real-world settings. A positive development in this regard is a project developed with input from World Health Organization (WHO) affiliated stakeholders, which aims to further map and describe traditional medicine (TM) specific research methods (88).

### 5.6 Mapping complexity in TCIM

Existing guidance on systematic reviews recommends the use of logical models as a pictorial way to communicate complex interrelationships. Logical models can be used as a tool for informing the systematic review program, including in particular the systematic review protocol (89). There are different types of logical models, and a taxonomy has been proposed by Rehfuess et al. (90).

**Figure 3:**
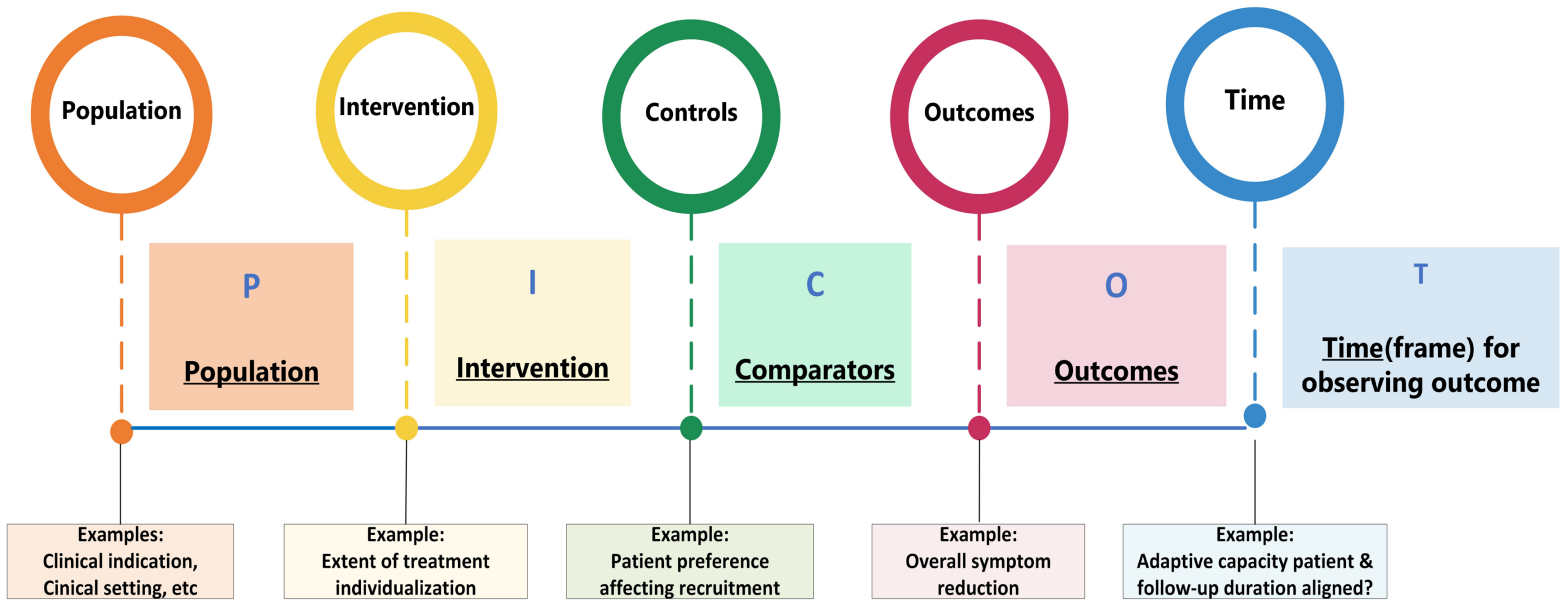
Mapping complexity in TCIM systems, aided by the PICOT framework.

TCIM modalities usually consist of a diverse yet distinct set of approaches for targeting a variety of outcomes. Moreover, therapeutic approaches as employed in routine clinical practice, are sometimes modified for methodological and/or practical purposes in RCTs. It is recommended that the relationships between different elements that are deemed likely to affect outcomes are mapped. For this purpose, use can be made of the ‘PICOT’ framework as commonly utilized in evidence synthesis (91, 92). Figure 3 describes the PICOT elements and gives some examples that are potentially relevant for TCIM modalities.

**Figure 4:**
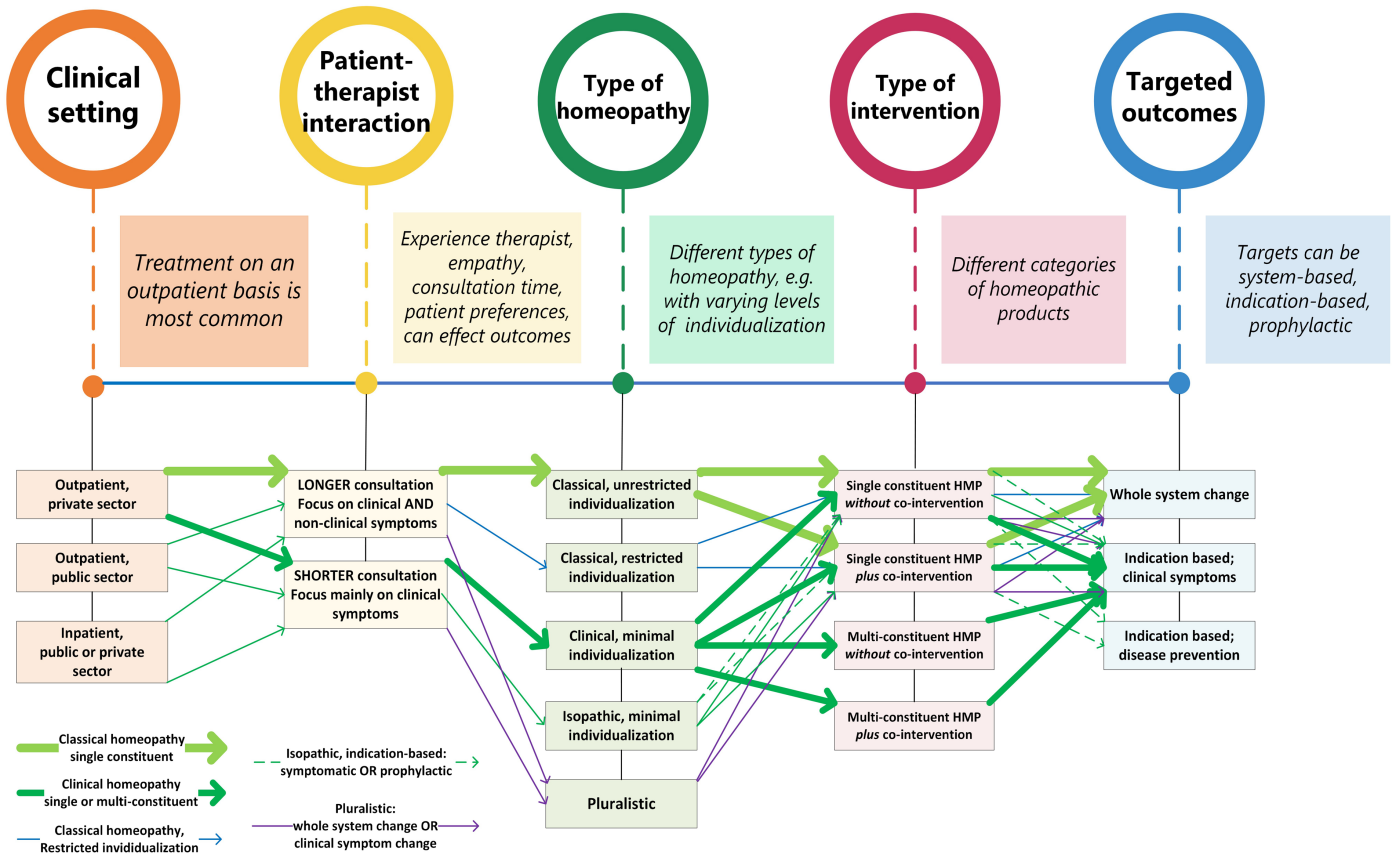
Visualizing complexity of the homeopathic treatment process. Thick arrows indicate the most common pathways.

TCIM specific PICOTs should be identified in a tailored manner, using as the main criteria those PICOT elements that are likely to influence the outcome(s) of the therapeutic process. The complex interactions between these elements should be visualized and explained. A detailed example of this complexity in homeopathy is depicted in Figure 4. The main purpose of this figure is to give an example how complexity can be visualized using PICOT elements. The main pathways are individualized (‘classical’) homeopathy with single HPs (thick, light green arrows) and non-individualized (‘clinical’) homeopathy with multi-constituent HPs (thick, dark green arrows). A detailed explanation of all the possible pathways is outside the scope of this article.

It should be emphasized that complexity can be mapped in a multitude of ways; the PICOT framework is only one of the possibilities that can provide some guidance and a possible structure for mapping complexity. In Figure 4 some, but not all, of the PICOT elements have been adopted. For instance, there is a lot of emphasis on the aspect ‘Intervention’, but little or no reference to the dimensions ‘Control’ and ‘Time’ respectively.

This approach contains some of the elements of a system-based logical model (90); in particular, the complexity of TCIM modalities as whole medical systems is mapped in relation to various targeted outcomes. For many TCIM modalities, including homeopathy, this will require a focus on mapping the complexity of the intervention, including aspects such as treatment individualization, etc.

### 5.7 Conceptual framework on possible mode of action; the example of homeopathy

Theoretical considerations on why, how, and under which circumstances an intervention works or does not work, are important in systematic reviews that aim to incorporate a complexity perspective (79). Most TCIM systems have evolved based on observations in clinical practice, which is why, as explained earlier, a ‘reverse research strategy’ has been used (51). Therefore, there will often be limited knowledge on the ‘mode of action’ in a biomedical sense. Nonetheless, all TCIM systems are underpinned by several implicit and explicit (ontological) assumptions about how the world we are researching works. Therefore, an attempt should be made to describe these theoretical considerations, and any available data that support them. We illustrate this by the example of homeopathy.

Hahnemann, the founder of homeopathy, introduced the concept of a biphasic action of treatment with HPs. The primary action is the direct effect of the HP on the organism; the secondary action is the adaptive reaction of the organism to the primary effect of the HP (93). The intended therapeutic effect is associated with the secondary action. In this conceptual frame, homeopathy belongs to the broader class of regulative medicine procedures, which rely on triggering adaptive reactions in the organism. Effects of regulative medicine procedures are probabilistic rather than deterministic. The probability of success of HPs can be increased by maximizing the similarity of the symptoms of the patient to those that the HP can induce in healthy subjects.

A leading conceptual framework for the mode of action of HPs is an ‘information model’ in which the cause for the medical effects of HPs arises from an adaptive restorative action *within* the organism, as a response to some kind of information received in the course of the homeopathic therapeutic process (94).

Physicochemical investigations have revealed empirical evidence for specific properties of HPs (95, 96) to be related to some kind of structure within HPs that could carry specific information. Multiple theories and models have been proposed to explain the possible mode of action of HPs (97). However, it should be acknowledged that none of these models is currently supported by sufficient or conclusive experimental evidence. Also, fueled by a perceived lack of plausibility, the effects of HPs are controversially debated (98).

A complex systems framework predicts that context factors can affect outcomes, and some examples from well-established basic research models are given below. For instance, biological effects of HPs can be nullified after exposure of HPs to pulsed microwaves (99), which suggests that the information carrying structure can be disturbed by electromagnetic radiation (100). Other preclinical experimental studies also indicate that environmental factors can modulate the effects of HPs: effects of HPs of gibberellic acid or *Argentum nitricum* on growth of *Lemna gibba* (101, 102) were dependent on gibbosity of duckweed which is induced in nature by seasonal factors; effects of HPs of thyroxine on the metamorphosis of *Rana temporaria* were dependent on the ecotope (altitude) of the animals (103); effects of HPs of *Mercurius corrosivus* on the growth rate of mercury-stressed *Lemna gibba* were dependent on the stress level (104, 105); and effects of HPs of gibberellic acid on *Pisum sativum* (106) or *Hordeum vulgare* (107) were modulated by seed lot quality as related to cultivation history.

These findings are consistent with a complex systems perspective in which effects of HPs are indirect and modulated by the influence of the broader environment.

### 5.8 Implications for the systematic review protocol template

The HOMA project plans a series of systematic reviews based on a range of health conditions. In order to avoid duplication of effort, a detailed protocol template has been developed (as published on the Open Science (OSF) preprint server: https://osf.io/preprints/osf/zpyhf_v2 and as accepted for publication in the journal ‘Systematic Reviews’). As indicated in Figure 1, this protocol template will be used as the basis for writing the indication-specific study protocols. It incorporates the use of current published ‘gold standard’ guidance including the Cochrane Handbook for Systematic Reviews of Interventions, risk-of-bias tools (RoB 2 for RCTs and ROBINS-I for non-randomized studies), the GRADE framework (including Summary of Findings tables), PRISMA-P, PRISMA 2020, as well as an ‘intervention Complexity Assessment Tool for Systematic Reviews (iCAT_SR). When not fully appropriate, such as for instance in the case of the iCAT_SR, existing guidance has been adapted for our purposes to the minimum required extent. In one instance, we make use of a specific tool that assesses how well the studies are aligned with the principles and practice of homeopathy (Model Validity of Homeopathic Treatment tool) to address a gap in published guidance.

Due to the likelihood of many similarities, this protocol template can also serve as a basis for systematic reviews of other TCIM modalities that aim to adopt a complex systems perspective. We elaborate on some of the key aspects below.

#### 5.8.1 Inclusion of both RCTs and NRSIs

It has been observed that due to a strong value preference, patients seeking homeopathic treatment are often unwilling to participate in clinical trials (108). In whole medical systems, patients are active participants in their own healing process, and it is likely that patients who consent to participate in RCTs, are not fully representative of patients that are seen and treated in ‘real-world’ settings. This leads to a selection bias and potentially affects treatment outcomes, as observed for instance in anthroposophic medicine (109). Differences between patients included in homeopathic clinical trials and those seen in real-world settings, can lead to high levels of ‘indirectness’ (110). NRSIs can complement RCT data not only in TCIM; Conventional drug treatments are also increasingly individualized and due to the related shrinking size of target populations, the assessment of both sources of evidence is gaining importance in many areas of medical research (111, 112). RCTs and NRSIs complement each other and both are needed to obtain a comprehensive overview of the literature in line with the PRISMA guidance on complex interventions (113).

#### 5.8.2 Literature search

To mitigate and better assess the risk of a reporting bias (114), the literature search will be particularly comprehensive, including gray literature, theses, pre-publication server and trial registries. Authors will be contacted for missing information.

#### 5.8.3 Multiple health conditions

Comparing evidence across multiple health conditions using the same set of methods allows for assessing the consistency of findings. This approach can help develop theories on why differences arise—whether due to variations in mechanisms of action, placebo effects, study quality, or measurement instruments. It may also help to determine whether effect sizes remain similar despite differences in disease pathophysiology, whether variations in effectiveness are linked to individual patient characteristics, and whether the effects are mainly restricted to particular health conditions. The ultimate selection of health conditions was informed by a combination of pragmatic and methodological reasons, including clinical relevance and data availability.

#### 5.8.4 Complexity

As explained, investigating complexity is crucial for improving the understanding of when, why, how, and under what circumstances TCIM interventions succeed or fail.

In line with Cochrane Handbook Chapter 17, which provides guidance on addressing complexity in systematic reviews (115) we operationalize implementation of complexity via use of the ‘Intervention Complexity Assessment Tool for Systematic Reviews’ (iCAT_SR), which situates interventions along a continuum from simple to complex (82). It accounts for factors such as the number of components, degree of individualization, and contextual influences. Although iCAT_SR is typically applied at the review level, we propose that for TCIM modalities, it is adapted for use at the level of individual studies, similar to the approach taken by Viswanathan et al. (116). This approach facilitates capturing complexity at the level of the specific clinical indication rather than for the TCIM modality as a whole. In the HOMA project, specific domains have been selected and tailored to homeopathy (see protocol template: https://osf.io/preprints/osf/zpyhf_v2). The adapted use of iCAT_SR serves a dual function: (A) it describes the complexity and (B) it supports subsequent synthesis by enabling comparisons across indications and informing analytical strategies.

#### 5.8.5 Quantitative synthesis

To better understand the independent and combined effects of intervention components—and whether these are context-dependent, time-sensitive, or non-linear—subgroup analyses and meta-regressions will be conducted. These analyses will compare individualized versus non-individualized interventions, single versus complex homeopathic preparations, and investigate indication-specific contextual factors (e.g., co-therapy, comorbidity), as well as selected domains from iCAT_SR.

From a complex systems perspective a multiplicity of effects is expected (62). To address the potential multiplicity of effects, a range of outcome measures will be included, spanning both health-related as well as broader, patient-centered outcomes such as treatment satisfaction and overall perceived effectiveness. Long-term effects reported in NRSIs will be considered to capture potential emergent properties of the intervention–system interaction. Where appropriate, multilevel models may be used to account for correlations among outcomes and the hierarchical structure of the data. This strategy aligns with established approaches for synthesizing quantitative evidence in the context of complex interventions (117).

#### 5.8.6 Model validity and external validity

In addition to measuring internal validity using instruments such as ROB-2 and ROBINS-I, external validity, representing the generalizability of findings, plays an important yet often neglected role (118). For whole medical systems, it has been argued that model validity represents a third relevant component, reflecting the concordance between the trial study design and the “state of the art” practice for the intervention (119). External validity will be assessed using the RITES tool (120), and alignment with the principles of homeopathy (model validity) will be assessed using the MVHT tool as developed by Mathie et al. (121).

#### 5.8.7 GRADE

The GRADE (Grading of Recommendations, Assessment, Development, and Evaluations) approach provides a structured framework to assess the certainty of evidence by rating the domains risk of bias, inconsistency, indirectness, imprecision, and publication bias (122). Estimating the certainty of evidence poses a challenge for complex interventions, as they are inherently heterogeneous and may therefore lead to a biased representation through GRADE, due to downgrading in the domains of imprecision and inconsistency (123). For this reason, in the HOMA project we will apply a non-contextualized approach to GRADE (124) that assesses the certainty of evidence in a non-null effect of homeopathy compared to controls, as has been suggested for complex interventions (123). Given the expected contextual variability and heterogeneity, using the null effect as the operative threshold allows us to examine whether evidence consistently supports the presence of an effect across contexts, before considering questions of magnitude and clinical relevance.

#### 5.8.8 Involvement of consumers

Patient involvement took, and will take, place at two levels. A patient advocate with experience in evidence-informed health care, systematic reviews, patient engagement and complementary medicine helped inform this framework publication as well as the protocol template for the systematic review program. The patient advocate will also engage in the involvement of patient representatives during the work program, provide input into the conclusions of the series of systematic reviews as well as plain language summaries, and contribute to the dissemination of findings.

At the second level, ‘patient advisors’ with living experience of the interventions under investigation for specific health conditions will provide feedback on the indication-specific protocols and plain language summaries of the systematic review findings. Multidisciplinary teams for each review will identify these patient representatives.

Overall, the aim is to ensure integrated knowledge transfer throughout the program.

## 6 Complementarity of opposites: a key aspect of the framework

Complementarity between mutually incompatible concepts appears to be a fundamental principle in nature. Some examples are the particle-wave characteristics of light in physics, the complementarity between internal and external validity in epidemiology, and between reductionism and holism in epistemology.

Why is the parallel use of opposite concepts a key aspect of the framework for evidence synthesis in TCIM? Philosophically, it is related to the importance of integrating and framing the epistemic reductionism implicit in the evidence synthesis process within the holistic ontology of whole medical systems. Methodologically, it implies that the RCT as the preferred research method for assessing the certainty of evidence using GRADE, needs to be complemented by a broader and non-hierarchical (125) approach to evidence (54). The latter is also addressed in Table 1 and in the section on the implications for evidence requirements in TCIM. Since these methodological approaches are distinct, they need to be applied sequentially; step 1 considers the TCIM modality as a whole medical system with its paradigms, philosophical understanding and utilization; step 2 involves systematic reviews of the RCT and NRSI data; and in step 3 the implications for the TCIM system are assessed, based on the systematic review data together with other evidence types and further considerations. Step 3 involves the contextualization of the available evidence, for which different frameworks have been proposed, the main ones being the ‘evidence-to-decision’ framework (126, 127) and the ‘evidence use ecosystem analytical framework’ (53, 128). To reiterate, on the one hand, it is necessary to look at the available data on TCIM systems with a ‘narrow and focused lens’, using among others GRADE. On the other hand, it is essential that these findings, together with other data are re-integrated into an enriched understanding of the TCIM system as a whole.

Despite the apparent contradiction between complementary concepts, a deeper analysis reveals that one cannot function without the other. Advancement of knowledge is not possible without the tension between apparent opposites, e.g., in science the way to certainty is achieved by embracing doubt. For a fuller discussion of this principle, also referred to as the ‘coincidence of opposites’, see McGilchrist (129). In conclusion, the apparent contradiction between the opposites of holism and reductionism is creative and positive if the reductionistic component serves the purpose of contributing to a fuller understanding of the whole system.

## 7 Discussion

Proper framing of systematic reviews is essential for establishing a reliable evidence base and for supporting an informed societal discussion on the utility of TCIM. In this regard, the HOMA project can serve as an illustration of how a solid foundation for informing decisions and treatment recommendations can be created.

The HOMA systematic review program addresses an existing gap in indication-based systematic reviews of homeopathy by applying a more extensive literature search strategy and by incorporating both RCTs and NRSIs. Also, to the best of our knowledge, the HOMA project is the first homeopathic systematic review program that explicitly adopts a complex systems perspective. In addition to evaluating the efficacy, effectiveness, and harms of HPs, there is a need to better understand the contextual factors of homeopathy as a whole medical system within the framework of systematic reviews. This approach is borrowed from public health systems research, and its value for homeopathy will need to be demonstrated during the project. For instance, a potential limitation is uncertainty regarding the potential of iCAT_SR to fully capture the complexity of homeopathic interventions. Key aspects, such as the nature of the patient–practitioner interaction or the HP selection process, are rarely reported in sufficient detail and may therefore elude systematic classification. This limits the interpretability of complexity assessments, particularly at the level of individual clinical indications. While certain patterns may emerge when data from multiple indications are synthesized, isolated reviews may be underpowered to detect meaningful complexity-related effects. As proposed for complex interventions, it may become necessary to pool data across reviews and complement the current approach with additional strategies, such as component-based analyses or network meta-analyses.

Despite these latter options, opportunities to analyse contextual factors, such as the patient-therapist relationship, are likely to be constrained by the nature and quality of reporting in the included studies. Many homeopathy trials were not designed with a complex systems perspective in mind, and it is therefore quite conceivable that complexity relevant aspects are poorly, or not at all, reported. In the latter scenario, the HOMA project will serve as a ‘gap analysis’ and provide pointers to how the design of future trials can be improved.

A potential challenge could be the assessment of a non-null effect when the effect size is close to zero and potentially deemed to be trivial. The interpretation of small effects is complicated further by various factors leading to an increased likelihood of smaller effects of TCIM modalities in RCTs as compared to NRSIs. The first factor is related to the need for individualization of treatment. In homeopathy, the patient will not be sensitive and therefore non-responsive to an incorrectly individualized HP. Unless sensitive patients are ‘pre-selected’ prior to randomization (130), this phenomenon is likely to lead to a reduced average effect in RCTs. A second factor is that due to a strong preference for TCIM, patients are unwilling to participate in RCTs, leading to the RCT population being less representative of patients seen in real world settings (108, 109). The potential impact of patient preference on treatment outcomes is not fully understood, but the available data suggests that receiving one’s preferred treatment positively affects outcomes (131). In TCIM systems, these effects may be further enhanced due to the active role that the patient plays in her/his healing process. Patient preference is therefore likely to negatively affect the effects in the TCIM arm of RCTs (because patients with a strong preference refuse to participate). A third factor is related to the effect of the patient-practitioner interaction which is an integral part of TCIM systems. TCIM researchers have suggested that these effects can be enhanced in TCIM modalities and that despite larger overall effects in RCTs, the ‘specific effects’ may be small. Even small specific effects can be therapeutically valuable because they may not only add to, but also synergistically boost contextual treatment effects, potentially leading to what has been described as the ‘efficacy paradox’ (132). This hypothesis is supported by a recent study (133) which compared outcomes of psychotherapy approaches under conditions ranging from highly experimental (efficacy orientated) to ‘real-world’ (effectiveness orientated). This study found that the ‘therapist effect’ tends to get enhanced when moving to the ‘effectiveness pole’ of the ‘efficacy-effectiveness continuum. Moreover, this study indicated that the likelihood of small effects increases when the focus is on components of the same treatment modality. This is likely to apply to many RCTs of TCIM treatments, which are often focused on separating the ‘non-specific’ component (effects due to patient-therapist interaction) from the ‘specific’ component (TCIM treatment effect). The above-mentioned factors, together with the contextual and implementation variability of TCIM systems as complex interventions (123), underline the inherent challenges associated with interpreting small effects.

The basis for the evidence synthesis in the HOMA project is limited to RCTs and NRSIs. Various authors justifiably argue that a broader mix of different types of research, both quantitative and qualitative, may be appropriate for TCIM modalities. As mentioned, a systems-based approach requires that RCT and NRSI evidence is complemented by other types of evidence, and to assess the coherence between multiple types of evidence. It has been argued that circular evidence models or matrix methods (54) could help address the limitations associated with an exclusive reliance on RCTs. However, the synthesis of different sources of evidence and their associated limitations has so far only been carried out in a few exemplary cases (134) or only for specific study types (135).

It is out of the scope of the HOMA project to systematically look at other types of quantitative and qualitative data in the indication-based systematic reviews. However, the project will also conduct two systematic reviews of the commonly used HPs Arnica and Arsenicum album. In addition to RCTs and NRSIs, complementary types of evidence will also be included, by applying a ‘mixed-method’ approach (86).

Complexity is a relevant feature in many healthcare interventions, but its nature and implications vary depending on the system in question. While biomedical interventions can also exhibit complexity—such as multiple interacting components, varying implementation strategies, or context-sensitive effects—these are often confined to specific treatments or care pathways. In contrast, TCIM whole medical systems are characterized by systematic individualization, an integrated and relevant patient–practitioner interaction, and adaptability across a broad range of indications. Its complexity is not merely an attribute of specific interventions but is ontologically embedded in its underlying therapeutic logic. Therefore, understanding the effects of TCIM whole medical systems requires a broader conceptual and methodological approach that captures system-level interactions and emergent properties beyond isolated components. Some progress in the TCIM sector has been made in this regard; Graham et al. (136) propose a ‘Complexity Informed Implementation Model’ which emphasizes the intervention as a ‘catalytic probe’ and as part of a cyclical process involving the observation of the change process, evaluation of outcome based on pattern recognition and adjustment of the intervention as appropriate.

A complex systems perspective places a lot of emphasis on the importance of contextual factors. While making treatment recommendations and/or the formulation of treatment guidelines is outside the direct scope of the HOMA project, integrating the evidence into a broader context, considering factors such as the balance of benefits and harms, patient values and preferences, cost-effectiveness, and real-world feasibility is essential. As mentioned, this contextualization can be based on the evidence-to-decision framework (137), an evidence ecosystem analytical framework (53) or similar approaches. It is highly compatible with TCIM principles to consider the patient perspective (138) and the challenges of drawing coherent conclusions from the evidence synthesis of different study types.

As pointed out in this article, there are many ontological similarities between homeopathy and other TCIM systems. The proposed framework is therefore likely to be useful for the broader TCIM field, even though tailoring to the particularities of each TCIM modality will be required. The HOMA systematic review program aspires to be an example that systematic review authors of TCIM modalities can use as a point of orientation and for informing decisions if pragmatic adaptations are necessary. The HOMA project also hopes to inform how the epistemic appropriateness of RCTs and NRSIs for the evidence base of TCIM systems can be further improved.

Appropriate framing of systematic reviews and systematic review programs is essential and contributes to a balanced societal discussion about the role of TCIM in healthcare systems.

Evidence synthesis of TCIM systems can move beyond conventional approaches by framing evidence within its complexity and context, together with real-world data and patient perspectives. This approach entails methodological challenges and will require structured gap analyses to guide future research and improve applicability for public health and individualized patient care (139).

## Footnotes

1
https://osf.io/preprints/osf/zpyhf_v2

2
www.tcih.org
